# Proteomic and immunochemical approaches to understanding the glycation behaviour of the casein and β-lactoglobulin fractions of flavoured drinks under UHT processing conditions

**DOI:** 10.1038/s41598-018-28943-4

**Published:** 2018-08-27

**Authors:** Ovidiu I. Geicu, Loredana Stanca, Anca Dinischiotu, Andreea I. Serban

**Affiliations:** 1 Department of Biochemistry and Molecular Biology, Faculty of Biology, University of Bucharest, 91-95 Splaiul Independentei, 050095 Bucharest, Romania; 2 Department of Preclinical Sciences, Faculty of Veterinary Medicine, University of Agronomic Sciences and Veterinary Medicine of Bucharest, 105 Splaiul Independentei, 050097 Bucharest, Romania

## Abstract

Dairy technology used to produce sweetened milk products might introduce additional advanced glycation end products (AGEs) into the diet. These molecular messengers are linked to detrimental health effects. Using a model accurate to the thermal treatment, reducing sugars, main protein content, and prolonged storage of ultra-high-temperature-sterilized (UHT) milk, we studied the behaviour of milk proteins during glycation. Two-dimensional electrophoresis (2-DE) profiles and western blots of glycated total casein revealed the major contributions of α_s2_-casein and β-casein and the relatively minor contributions of κ-casein towards the formation of N^ε^-carboxymethyllysine (CML)-positive aggregates. Glycated κ-casein had the lowest furosine (FUR), 5-hydroxymethylfurfural (HMF) and AGEs content. Conversely, the α-casein fraction demonstrated a high susceptibility to glycation, having the highest FUR, HMF and AGE levels. The gel-filtration elution profiles and the corresponding fraction fluorescence revealed that glycated casein aggregates were highly fluorescent, while the β-lactoglobulin glycation profile was similar to that of bovine serum albumin, and fluorescence was detected mainly in tetramers. Although CML is not a cross-linking AGE, it was only detected in large molecular aggregates and not in glycated monomers. Our results also indicate that in casein, glycation-induced changes in the UHT conditions were less deleterious than the subsequent 90 day storage period.

## Introduction

Children prefer sweet foods due to their high palatability rating^1^, including flavoured milk drinks, which contribute to a considerable proportion of total dairy consumption. Flavoured milk contains both natural sugar (lactose) and added sweeteners, such as sucrose, glucose-fructose syrup, or non-caloric sweeteners, depending on the brand. Each manufacturer may use a unique formula, with varied amounts and types of added sweeteners. Concerns regarding the detrimental health effects of added sugar have led to potential food reformulation strategies^2^.

In modern dairy technology, product safety and extended shelf-life are ensured by heat treatments such as moderate temperature pasteurization (65 °C, 30 min), high-temperature pasteurization (72 °C,  15 s), ultra-high-temperature (UHT) sterilization (130–140 °C, 3–8 s) and in-container sterilization (112 °C, 15 min)^3^. As a consequence of the heating treatments, the proteins, carbohydrates and vitamins in the milk undergo chemical and biochemical changes^4^. In flavoured UHT milk products, these changes can be exacerbated by the addition of reducing sugars and the subsequent storage of these milk products at room temperature. Some of these changes include: the degradation of lactose to lactulose and acids; the degradation of lateral chains of amino acids through β-elimination with the formation of dehydroalanine, a compound that can readily react with lysine yielding lysinoalanine; the formation of disulphide-linked complexes of β-lactoglobulin (β-Lg) with κ-casein; and the denaturation of whey proteins^3,5^. In addition, alterations may occur also via the Maillard reaction, which begins when the free amino groups of a protein react non-enzymatically with reducing sugars to form Schiff’s bases, which are temporarily stabilized through Amadori rearrangements. The best known Amadori products formed via the Hodge pathway are fructoselysine (when the reducing agent is a hexose) and lactoselysine (when the reducing agent is lactose). Subsequently, a range of other reactions take place, including cyclizations, dehydrations, retro aldolizations, oxidations, rearrangements and condensations, which finally give rise to AGEs with various chemical structures such as: CML^6^, pentosidine^7^, glucosepane^8^, crossline^9^ and melanoidin^10^, as well as brown nitrogenous polymers and co-polymers known as melanoidins^11^. One of the best characterized AGEs is CML, formed by the reaction of glyoxal and lysine or by the oxidative cleavage between C-2 and C-3 of fructoselysine. CML is colourless, non-fluorescent, and does not cross-link proteins. Because of its charge density, CML is located on the surface of glycated proteins and is thought to be the predominant antigen recognized by anti-AGEs antibodies^12^. CML is a stable product and was proposed as a marker of the nutritional quality of heat-treated foods^13^.

Diet is a major environmental source of proinflammatory AGEs and their impact on human health remains unclear^14,15^. Two-thirds of orally-absorbed dietary AGEs (10% of the amount ingested) are retained in bioreactive forms in human tissue and the rest is removed by renal excretion^16^. It seems that dietary AGEs, together with endogenous AGEs, may promote systemic glycoxidant burden, oxidative stress and cell activation by the receptors of AGEs (RAGE), enhancing the vulnerability of AGEs-accumulating tissues to diabetic-like injury^17,18^. Therefore, in addition to the effects of added sugars on children’s health, the intake of AGEs and their effects on children’s health are important and concerning aspects of the consumption of flavoured milk products

The aim of this study was to investigate the degree of milk protein modifications by the Maillard reaction using experimental conditions that simulate the processes and prolonged storage (a typical recommended shelf life of approximately 3 months) of flavoured UHT milk products. Importantly, the ratio of the main protein constituents, α-, β- and κ-caseins (Csn) and β-Lg, to reducing sugars was consistent with Romanian brands of standardized milk or flavoured UHT milk. Previous studies investigating Maillard products formation in food have used conditions more severe than those used in food processing procedures (such as higher reducing sugar concentrations, different protein to reducing sugar ratios and longer exposures to high temperatures). Therefore, the monitoring of heating treatments, the identity and quantity of added reducing sugars and the storage time are necessary when considering the quality of milk products. In this context, our data offer a relevant description of the state of milk proteins undergoing typical UHT treatment.

## Results and Discussion

### FUR and HMF quantification

FUR, HMF and lactulose, not present in unprocessed milk, can be used as protein heat damage indicators and to distinguish between UHT milk, pasteurized milk and in-container sterilized milk^19,20^. The FUR and HMF amounts obtained in our experimental setup are shown in Table 1. The highest FUR concentration (212 mg/100 g protein) was obtained in UHT-treated α-Csn, glycated in the presence of the reducing sugar mix, which showed that the α-Csn fraction was the most sensitive to glycation. Lysine content differs among the casein types (14, 24, 11 and 9 residues for α_s1_-, α_s2_-, β- and κ-Csn, respectively)^21^ and the number of lysine residues in the α-Csn fractions explain the high FUR concentration of the α-Csn fraction. In addition, other studies of UHT milk protein fractions using mass spectrometry (MS) have identified fewer lactosylation and hexosylation reactive sites than the total possible sites. The hexosylation and lactosylation reactive sites overlapped in UHT milk and were specific to each casein fraction, as described by Milkovska-Stamenova and Hoffmann: 8 lactosylated to 1 hexosylated site in α_s1_-Csn, 15 lactosylated to 4 hexosylated sites in α_s2_-Csn, 4 lactosylated to 1 hexosylated site in β-Csn and 6 lactosylated to 1 hexosylated site in κ-Csn^22,23^. However, our results showed a similar FUR content between β-Csn and κ-Csn subjected to UHT treatment in the presence of lactose and slightly increased FUR for UHT β-Csn samples glycated with the reducing sugars mix (Table 1). These data suggest that under UHT conditions, β-Csn and κ-Csn have comparable sensitivity towards lactosylation and hexosylation, β-Csn being slightly more susceptible. In the case of β-Lg from UHT milk samples, 12 lactosylated to 6 hexosylated sites from 15 possible sites were identified^22,23^, which is in agreement with the high 118 and 220 mg of FUR/100 g protein we detected (Table 1) in UHT β-Lg in the presence of lactose or reducing sugar mix, respectively. The FUR and HMF concentrations (Table 1) we detected in UHT samples with lactose were similar to those obtained in UHT milk samples^19,20^. By contrast, UHT samples incubated with the reducing sugar mix had generally higher FUR and HMF concentrations than what has been described in UHT milk but lower than those reported in lactose-free (hydrolysed lactose) UHT milk^19,20^.

**Table 1:** Glycation markers in milk protein samples.

Milk proteins (MP) and reducing sugars (S) treatment	Glycation markers (Gmk)				
	FUR (mg/100 g protein)	HMF (μg/100 mL)	AGEs (μg/mg protein)	RFU λ_Ex_ 335 nm/λ_Em_ 410 nm	RFU λ_Ex_ 370 nm/ λ_Em_ 440 nm
TCsn control, UHT	ND*	ND*	11 ± 0.6	33.1 ± 8.4	25.2 ± 2.2
TCsn, Lac, UHT	97.1 ± 7.4	4.2 ± 0.4	77 ± 6.9	92.3 ± 7.4	58.8 ± 6
TCsn, Lac, Glc-Fru, UHT	205.2 ± 13	7.5 ± 0.4	267 ± 13	200.6 ± 11.2	148.4 ± 11
TCsn control, non-UHT	ND*	ND*	10 ± 0.8	32.2 ± 4.2	24.2 ± 3.1
TCsn, Lac, no UHT	82.49 + 5.6	ND*	54 ± 4.3	85.5 ± 4.5	50.8 ± 2.7
TCsn, Lac, Glc-Fru, non-UHT	133.8 ± 9.1	ND*	117 ± 8	157.3 ± 9.2	102.7 ± 7.7
α-Csn control, UHT	ND*	ND*	14 ± 1.2	35.3 ± 4.1	28.4 ± 1.4
α-Csn, Lac, UHT	105.1 ± 9.3	5.5 ± 0.25	116 ± 8	149.5 ± 12	107.4 ± 7.8
α-Csn, Lac, Glc-Fru, UHT	212.1 ± 15	8.9 ± 0.37	459 ± 21	270.9 ± 14	205.0 ± 13
α-Csn, Lac, Glc-Fru, non-UHT	143.10 ± 12	ND*	254 ± 16	198.2 ± 15	142.5 ± 11
β-Csn control, UHT	ND*	ND*	16 ± 1.3	33.4 ± 2	26.6 ± 3.1
β-Csn, Lac, UHT	86.8 ± 4	3.1 ± 0.11	45 ± 3.9	76.8 ± 4.6	55.0 ± 3.2
β-Csn, Lac, Glc-Fru, UHT	180.6 ± 9	6.7 ± 0.41	190 ± 11	162.1 ± 12.1	128.6 ± 8.6
β-Csn, Lac, Glc-Fru, non-UHT	115.9 ± 8.4	ND*	87 ± 5.1	109.8 ± 9.9	80.9 ± 4.4
κ-Csn control, UHT	ND*	ND*	15 ± 1.2	32.2 ± 1.3	26.6 ± 1.3
κ-Csn, Lac, UHT	82 ± 3.4	2.7 ± 0.17	43 ± 1.5	72.7 ± 2.2	42.9 ± 3.3
κ-Csn, Lac, Glc-Fru, UHT	150.1 ± 13	5.6 ± 0.21	161 ± 11	147.7 ± 8.7	111.5 ± 8.8
κ-Csn, Lac, Glc-Fru, non-UHT	104.4 ± 7.8	ND*	59 ± 3.2	92.1 ± 6.6	71.2 ± 5.5
β-Lg control, UHT	ND*	ND*	13 ± 1.2	30.6 ± 1.3	21.4 ± 1.2
β-Lg, Lac, UHT	118.0 ± 9	2.5 ± 0.2	108 ± 8	129.7 ± 8.7	102.5 ± 7.6
β-Lg, Lac, Glc-Fru, UHT	220.0 ± 14	5.4 ± 0.3	421 ± 21	222.5 ± 12	142.7 ± 12
β-Lg control, non-UHT	ND*	ND*	12 ± 1.2	29.8 ± 3	19.8 ± 1.1
β-Lg, Lac, non-UHT	56.5 ± 5	ND*	69 ± 4	70.9 ± 4.5	49.2 ± 2.3
β-Lg, Lac, Glc-Fru, non-UHT	102.0 ± 8.7	ND*	134 ± 7	102.3 ± 5.6	70.2 ± 4.4

FUR and HMF were analysed by HPLC. FUR is indicative of compounds formed early in the glycation process, while HMF is an intermediate glycation compound. All experiments were performed in triplicate and FUR, HMF and AGEs data are shown as the mean of the absolute values ± the standard deviation (n = 3). *ND – not detected.

The influence of 55 mM glucose-fructose and UHT treatment on FUR and HMF formation were highlighted using the two ratios *R*_*s*_ and *R*_*UHT*_, which are shown in Table 2 and were calculated from data shown in Table 1. Ratios close to 1 show that the reducing sugar mix or UHT conditions contribute to a lesser extent to the glycation process. In UHT total caseins (TCsn), including α-Csn and β-Csn, the addition of the glucose-fructose mix induced a 2-fold increase in FUR content compared with that in the UHT κ-Csn and β-Lg samples glycated with lactose alone, which had an approximately 1.8-fold increase in *R*_*s*_ (Table 2). This suggests that lysine residues in the κ-Csn and β-Lg proteins are more sensitive to lactosylation rather than hexosylation. Interestingly, in the β-Lg samples, the *R*_*s*_ ratio was very similar for UHT and non-UHT conditions (Table 2), which revealed the high glycation susceptibility of β-Lg in the presence of the glucose-fructose mix.

**Table 2:** The relative contribution of the reducing sugar mix (*R*_*S*_) and UHT treatment (*R*_*UHT*_) to the glycation marker (Gmk) levels.

Type of MP and thermal treatment	***R***_***S***_^***a***^				Type of MP and reducing sugars	***R***_***UHT***_^***b***^		
	FUR	HMF	AGEs	RFU		FUR	AGEs	RFU
TCsn, UHT	2.11^**^	1.79^*^	3.47^***^	2.17^**^	TCsn, Lac	1.18	1.43	1.08
TCsn, non-UHT	1.62^*^	—	2.17^**^	1.84^*^	TCsn, Lac, Glc-Fru	1.53^*^	2.28^**^	1.30
α-Csn, UHT	2.02^*^	1.62^*^	3.96^***^	1.81^*^	α-Csn, Lac, Glc-Fru	1.48	1.81^*^	1.37
β-Csn, UHT	2.08^*^	2.16^**^	2.92^**^	2.11^**^	β-Csn, Lac, Glc-Fru	1.56^*^	2.18^**^	1.48^*^
κ-Csn, UHT	1.83^*^	2.07^**^	3.74^***^	2.03^*^	κ-Csn, Lac, Glc-Fru	1.44	2.73^**^	1.60^*^
β-Lg, UHT	1.86^*^	2.57^**^	3.89^***^	1.70^*^	β-Lg, Lac	2.09^**^	1.58^*^	1.83^*^
β-Lg, non-UHT	1.80^*^	—	1.96^*^	1.44	β-Lg, Lac, Glc-Fru	2.16^**^	3.14^***^	2.16^**^

Samples glycated with 115 mM Lac and those detected in non-UHT conditions were used as references for *R*_*S*_ and *R*_*UHT*_, respectively (see Table 1 for mean values). ^*^P < 0.05, ^**^P < 0.01, ^***^P < 0.001 Student’s T test, two-tailed distribution, two sample unequal variance, n = 3. S – sugar content; MP – milk protein.

$${}^{{\rm{a}}}{R}_{s}=\frac{Gmk(MP+Lac+Glc+Fru)}{Gmk(MP+Lac)}\,{}^{{\rm{b}}}{R}_{UHT}=\frac{Gmk(MP+S)UHT}{Gmk(MP+S)nonUHT}$$.

The furosine ratio *R*_*UHT*_ ranged from 1.44 to 1.56 for all the casein samples glycated with the reducing sugar mix. The smallest calculated ratio was for the TCsn sample glycated with lactose alone, which revealed that storage at room temperature for 90 days greatly contributed to FUR formation in non-UHT conditions (Tables 1 and 2). UHT treatment of the β-Lg samples had the highest impact on FUR content, increasing FUR content by approximately 2-fold compared with that in the non-UHT counterpart (Tables 1 and 2). The effect of UHT conditions might be explained by the thermal denaturation of the globular protein that decreases its hydrophobicity index^21^ and accelerates the formation of Amadori compounds.

The UHT Csn and β-Lg had free HMF concentrations ranging from 2.5 to 8.9 µg/100 mL, similar to those reported in UHT milk^20^. The highest free HMF concentration was detected in UHT α-Csn glycated with the reducing sugar mix, while the lowest free HMF concentration was detected in κ-Csn and β-Lg. Free HMF was not detected in non-UHT samples, emphasizing the effect of temperature on dehydration reactions involved in HMF formation. The linear correlation between HMF and FUR content was positive and quite strong as we obtained correlation coefficients of 0.90 for casein samples and 0.65 for casein and β-Lg samples together. As the absolute values of FUR and AGEs were higher and the HMF levels were lower in β-Lg than in TCsn, it is possible that in β-Lg, the Amadori products were converted into AGEs via reaction pathways that bypassed HMF formation. The addition of glucose-fructose greatly impacted the concentration of free HMF (Table 2) in β-Lg samples (*R*_*s*_ ratio was 2.57), while their effect was moderate in α-Csn (*R*_*s*_ ratio was 1.62). This suggests that HMF levels may be suitable for the study of the effects of high temperature on whey protein exposed to hexose reducing sugars and on α-Csn exposed to lactose.

### Evaluation of total and fluorescent AGEs

Within the AGEs complex and heterogeneous group, the most widely used in the evaluation of Maillard-induced milk protein modification are the non-fluorescent and non-cross-linking CML and pyrraline, the fluorescent and cross-linking pentosidine, and non-fluorescent and cross-linking compounds such as glyoxal- and methylglyoxal-lysine dimers^24–27^.

As most AGEs chemical structures remain unidentified, the typical fluorescence excitation and emission wavelengths (λ_Ex_ 340–370 nm and λ_Em_ 420–460 nm) are important properties for characterizing Maillard products formed in foods, except for AGEs containing arginine, which present λ_Ex_ 320–335 nm and λ_Em_ 380–410 nm^28–30^.

The highest AGEs concentration and fluorescence were recorded when UHT casein fractions and β-Lg were glycated with the reducing sugar mix (Table 1). As previously observed for FUR, the α-Csn and β-Lg were the most susceptible to glycation, in correlation with their high numbers of lysine and arginine residues (14 and 6, respectively, for α_s1_-Csn; 24 and 6, respectively, for α_s2_-Csn; 15 and 3, respectively, for β-Lg)^21^. Important correlations were found between AGEs and fluorescence values recorded at both wavelengths (correlation coefficients were approximately 0.91), suggesting that a considerable part of the compounds recognized by anti-AGEs antibodies also have an associated fluorescence. The *R*_*s*_ ratio for the AGEs marker in the case of the UHT treated samples reached the highest value, ranging between 3 and 3.96 whereas for non-UHT treated samples the *R*_*s*_ was approximately 2 (Table 2), which suggests that the added reducing sugar and long storage period largely contributed to the formation of AGEs. The addition of 55 mM glucose-fructose caused the fluorescence ratio *R*_*s*_ of the UHT treated samples to increase (Table 2) to levels similar to those obtained by Bosch *et al*. in a comparative study between milk cereal-based infant food with or without the addition of 0.9% honey stored for 1–9 months at 25 °C^28^.

Our preliminary results, focused on the *in vitro* effects of glycated TCsn in enterocyte cells, revealed that a dose of 50 µg/mL AGEs induced oxidative stress and over-expression of RAGE and key inflammatory proteins, supporting pre-cancerous transformation^31,32^.

### Evaluation of milk protein aggregation and the associated fluorescence

**Figure 1:**
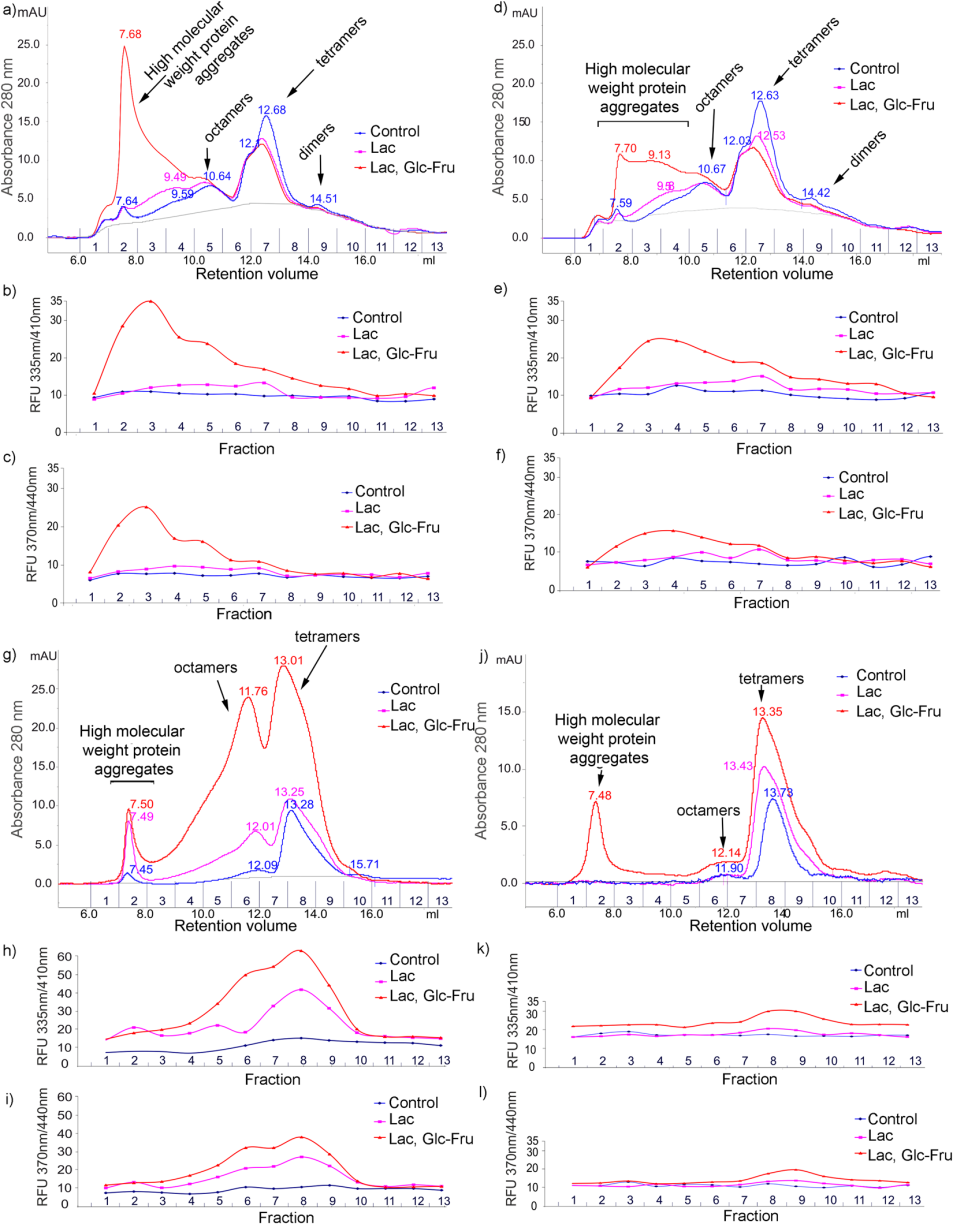
Representative FPLC chromatogram profiles of UHT total casein (**a**), non-UHT total casein (**d**), UHT β-lactoglobulin (**g**), and non-UHT β-lactoglobulin (**j**). Below each FPLC profile, the collected fraction fluorescence recorded at λ_Ex_ 335 nm/λ_Em_ 410 nm (**b**,**e**,**h** and **k**) and λ_Ex_ 370 nm/λ_Em_ 440 nm (**c**,**f**,**i** and **l**) are shown as relative fluorescence units (RFU). All experiments have been performed in triplicate. Legend: Control - no sugars were added, in blue; Lac – 116 mM lactose, in pink; Lac, Glc-Fru – 116 mM lactose and 55 mM glucose-fructose mix, in red.

**Figure 2:**
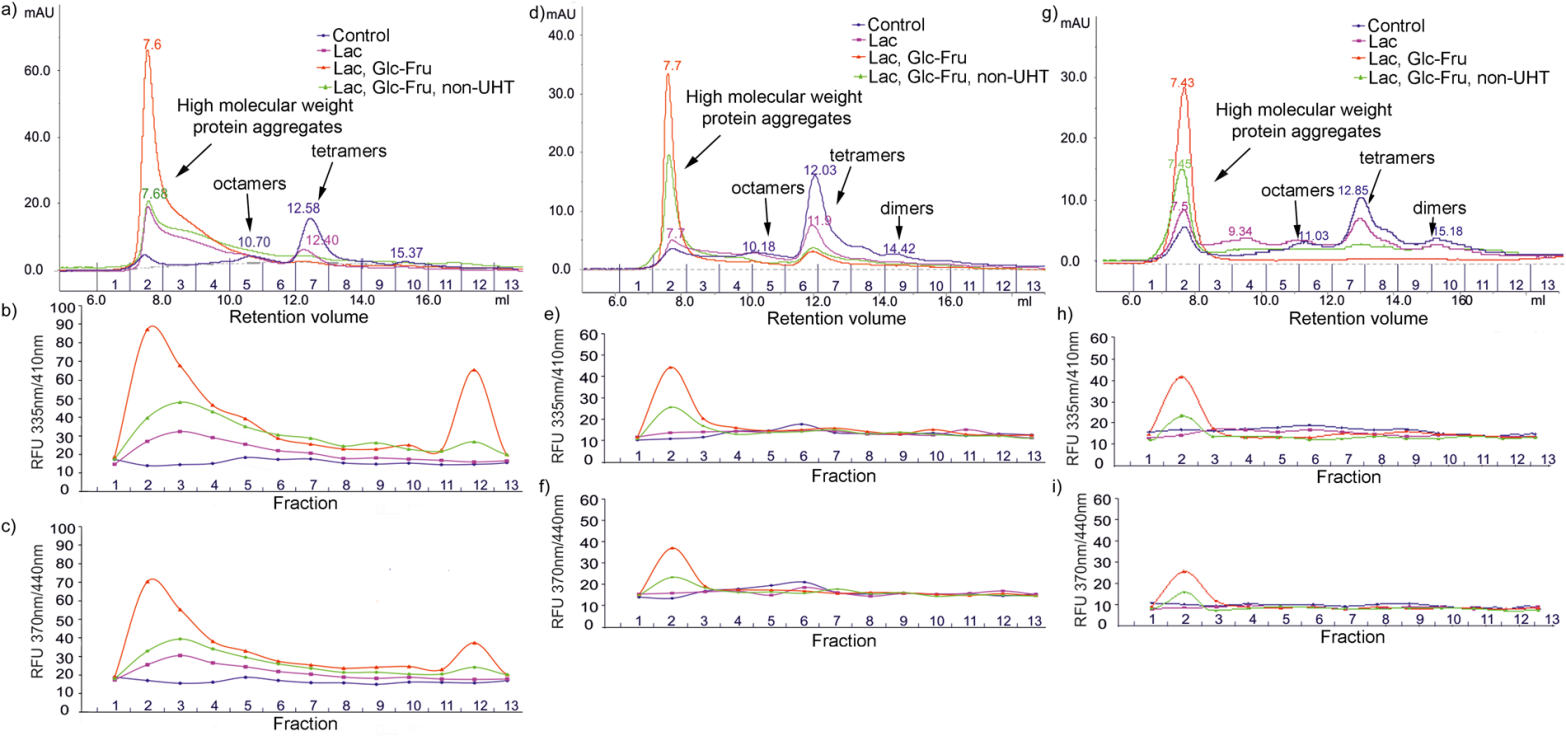
Representative FPLC chromatographic profiles of α-Csn (**a**), β-Csn (**d**) and κ-Csn (**g**) samples subjected to different conditions as described. Below each FPLC profile, the fluorescence of the collected fractions was recorded in RFU at λ_Ex_ 335 nm/λ_Em_ 410 nm (**b**,**e** and **h**) and λ_Ex_ 370 nm/λ_Em_ 440 nm (**c**,**f** and **i**). All experiments were performed in triplicate (n = 3). Legend: Control - no sugars added, blue; Lac – 116 mM lactose, purple; Lac, Glc-Fru – 116 mM lactose and 55 mM glucose-fructose mix, UHT, red; Lac, Glc-Fru – 116 mM lactose and 55 mM glucose-fructose mix, non-UHT, green.

The gel-filtration elution profile of control UHT-TCsn subjected to 90 day storage at room temperature was almost identical to that obtained in non-UHT conditions (Fig. 1a,d). The elution pattern of the control TCsn revealed the aggregation of caseins in dimers, tetramers, octamers and oligomers. The two highest overlapping UV-absorbent peaks at 12.6 and 12.03 mL retention volumes corresponded to the α-Csn/κ-Csn tetramers (Fig. 2a,g) and the β-Csn tetramers (Fig. 2d), respectively. Dimers were also observed at approximately the 14.5 mL retention volume, although they had low UV absorbance (Fig. 1a,d).

Due to a high proline content of fairly uniform distribution, all caseins possess a type of poly-proline helix structure and reduced content of α-helix or β-sheet structures^21^. The lack of highly organized structures probably explains the resistance of casein to denaturing agents such as heat, as well as their susceptibility to associate through hydrogen and hydrophobic bonds. At 4 °C, a β-Csn solution consists of monomers, while at 8.5 °C the monomers polymerize to form long thread-like chains of approximately 20 units. In addition, α_s1_-Csn polymerizes to form tetramers, and the degree of polymerization increases in a temperature- and concentration-dependent manner^21^.

In its unreduced form, κ-Csn consists largely of disulphide-linked polymers that exhibit hydrogen and hydrophobic bonds that contribute to the association with other κ-Csn and/or other caseins^21^. It is likely that the 4 M urea in the elution buffer used during the FPLC separation did not overcome the strong natural tendency of the caseins to associate, explaining the chromatogram profile.

The similar elution patterns of the UHT and non-UHT control TCsn samples revealed that the heat treatment was negligible to inducing protein cross-linking. Other studies have reported the formation of lysinoalanine and protein cross-linking under more extreme and prolonged heat treatments^25,33^ than those used in UHT milk production, which we reproduced.

The gel filtration patterns of the UHT and non-UHT TCsn glycated with lactose revealed tetramers decreased in 280 nm absorbance, concurrent with a slight increase in the molecular weight and UV absorbance of the oligomers (Fig. 2a,d). The slight increase in the molecular weight of the tetramer (fraction 7) corresponded to the increase in fluorescence, a phenomenon attributed to the formation of fluorescent intramolecular cross-linking compounds (Fig. 1b,c,e and f). The similarity between the elution profiles of the UHT and non-UHT TCsn samples glycated in the presence of lactose supports the idea that UHT treatment is less deleterious for caseins than is the 90 day storage period. More pronounced changes in the elution profile and fluorescence occurred when the UHT caseins were glycated in the presence of the reducing sugar mix, which revealed the formation of a high UV absorbance peak in the range of void volume (7.7 mL) corresponding to aggregates with high molecular masses and associated with a 3-fold increase in fluorescence (fraction 2–3) (Fig. 1a–c). The elution profiles of non-UHT TCsn glycated in the presence of the reducing sugar mix also showed aggregate formation. However, the associated fluorescence and UV absorbance were lower than those recorded for the UHT TCsn reducing sugar mix treated samples (Fig. 1d–f).

The β-Lg chromatograms and fluorescence fraction analysis revealed clear differences compared with the TCsn patterns (Fig. 1a,d,g and j). Although β-Lg has a globular conformation, its hydrophobicity is 5.03 kJ/residue, which is comparable to that of κ-Csn (5.12 kJ/residue)^21^. This could explain the tendency of the β-Lg monomer to associate into dimers, tetramers and octamers, which were observed in the non-UHT control sample (Fig. 1j). Notably, the tetramers had the highest UV absorption. The elution pattern of the control UHT β-Lg was largely similar to that of the non-UHT control sample; however it revealed a small proportion of heat-induced cross-linked protein aggregates. The chromatogram and fluorescence patterns of the non-UHT β-Lg samples glycated with lactose showed a slight increase in UV absorbance and fluorescence for the tetramer peak and an approximate 6 kDa increase in molecular weight (Fig. 1j–k). This mass increase could be attributed to the formation of Amadori products (0.2 kDa per Amadori product) and the intramolecular cross-linking of AGEs. The formation of Amadori products was confirmed by the quantification of FUR (Table 1). The elution and fluorescence profiles of the non-UHT β-Lg samples glycated in the presence of the reducing sugar mix (Fig. 1j–l) revealed the formation of a peak in the range of void volume (7.7 mL) corresponding to high-molecular-weight aggregates and increases in the UV absorbance and fluorescence of the tetramers and octamers. Interestingly, the molecular aggregate peak fluorescence was the lowest compared with those of the tetramers and octamers. Similar elution and fluorescence profiles were also noted for bovine serum albumin (BSA), when the glycated monomer, dimer and trimer peaks were found to be associated with UV absorbance and fluorescence increases and a slight increase in molecular weight^34–36^. The UHT β-Lg glycated with lactose or reducing sugar mix revealed a 2-fold increase in fluorescence and UV absorbance for the tetramer and octamer peaks and a slight decrease in the retention volume of the main protein peaks compared with that observed in the non-UHT samples (Fig. 1g–i). As in non-UHT conditions, β-Lg aggregates had low fluorescence levels. Caseins had a greater tendency to form fluorescent high-molecular-weight aggregates, whereas β-Lg aggregated to a lesser degree, forming tetramers, octamers and oligomers whose fluorescence surpassed that of the casein aggregates by almost 2-fold.

The control UHT α-Csn gel filtration elution pattern revealed four main peaks corresponding to dimers, tetramers, octamers and multimers with retention volumes of 15.37, 12.58, 10.70 and 7.7 mL (Fig. 2a). The chromatogram and the fluorescence profiles of the UHT α-Csn glycated with lactose revealed that the UV absorbance and the retention volume (12.40 mL) of the tetramer decreased while its molecular weight increased by 5–6 kDa. In addition, the peak (7.68 mL elution volume) UV absorbance and fluorescence increased. In the presence of the reducing sugar mix, the UHT α-Csn elution profile changed dramatically and revealed the formation of a huge molecular weight aggregate peak (7.6 mL retention volume) as well as the disappearance of the octamer, tetramer and dimer peaks. This aggregate was also associated with the highest UV absorbance and fluorescence (93 RFU recorded at λ_Ex335 nm_/λ_Em410 nm_) (Fig. 2a–c). Fractions 11–12 that correspond to α-Csn monomer were also fluorescent, a phenomenon that can be explained by intramolecular fluorescent compound formation. The elution and fluorescence profiles of non-UHT α-Csn incubated for 90 days at room temperature in the presence of the reducing sugar mix were similar to UHT samples except that the aggregate had half the UV absorbance and fluorescence (Fig. 2a–c).

The chromatogram profile of the UHT β-Csn glycated with lactose showed that the weight of the tetramers only increased by approximately 4 kDa while the fluorescence was lower than that of α-Csn (Fig. 2a–f). The gel filtration profiles of β-Csn exposed to the reducing sugar mix revealed that the tetramer peaks persisted, while the UV absorbance decreased. In addition, in similar conditions, the fluorescence and UV absorbance of β-Csn aggregates were half those of α-Csn (Fig. 2a–f).

The elution pattern of the control UHT κ-Csn samples showed that the aggregate peak had the highest UV absorbance compared with those of α- and β-Csn. This may have been caused by the detergent-like structure of κ-casein, crucial for protein association during micelle formation or through heat-induced protein cross-links. In addition to glycated tetramer and octamer peaks, the lactose-glycated κ-Csn chromatogram revealed decamers (9.34 mL retention volume). As with α-Csn, the gel-filtration pattern of UHT κ-Csn glycated with the reducing sugar mix showed the formation of a highly UV-absorbent and fluorescent protein aggregate peak as well as the disappearance of dimers, tetramers and octamers (Fig. 2g). Interestingly, in the glycated TCsn samples, the α- and κ-Csn tetramer peak, seen at 12.68 mL elution volume, seemed to be the most affected, as demonstrated by the diminished height of the peak (Fig. 1a,d), while the bulk of the β-Csn tetramer peak (12.1 mL elution volume) persisted. This effect was also observed when the separated casein fractions were analysed. The α- and κ-Csn tetramers completely disappeared, while β-Csn was just decreased (Fig. 2a,d,g).

### Evaluation of aggregate formation and the CML content of glycated milk proteins through SDS-PAGE and western blot analysis

**Figure 3:**
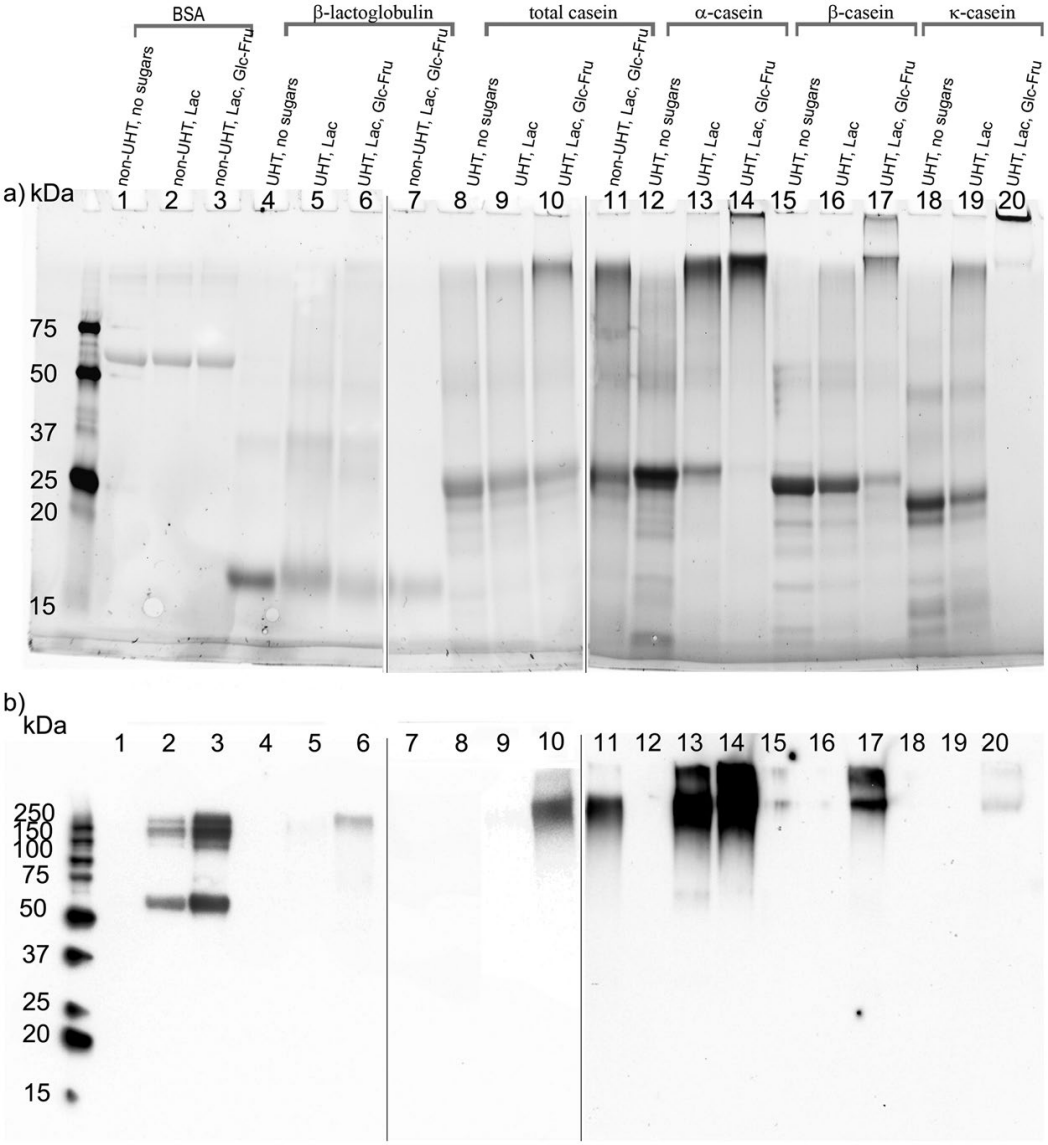
Representative SDS-PAGE gels (**a**) and CML blots (**b**) of BSA (lines 1–3), β-Lg (lines 4–7), TCsn (lines 8–11), α-Csn (12–14), β-Csn (15–17), κ-Csn (18–20). Line (1) is BSA in the absence of sugars, line (2) is BSA with Lac and line (3) is BSA with Lac and Glc-Fru. Except for BSA and the samples in lines 7 and 11, all other samples were UHT treated. No sugars were added to the samples in lines 4, 8, 12, 15 and 18 while in lines 5, 9, 13, 16 and 19 Lac was added to simulate UHT milk conditions. In lines 6, 10, 14, 17 and 20, the samples were glycated with Lac and Glc-Fru reducing sugar mix to simulate the conditions in flavoured UHT milk products. Lines 1–20 are shown in their entirety, however, the gel and corresponding blots were cropped to remove the unrelated lines, as designated by the white spaces left between lines 6–7 and 10–11. All the samples were stored at room temperature for 90 days and the experiments were performed in triplicate (n = 3).

To compare and understand the SDS-PAGE and the CML pattern of milk proteins, we used BSA samples (Fig. 3a, lines 1–3) subjected to the same conditions as non-UHT β-Lg as a reference. In the stronger denaturing and reducing conditions of SDS-PAGE (compared to gel filtration chromatography), we were able to reveal that the UHT control milk protein fractions were mainly in monomer form and associated into dimers to a lesser extent, demonstrating electrophoretic mobility (Fig. 3a, lines 4, 8, 12, 15 and 18). This suggests that the formation of cross-links is not driven by UHT treatment alone. β-Lg was the most mobile of the proteins; its monomer was localized at approximately 18 kDa and the dimer at approximately 37 kDa (Fig. 3a, line 4). The control UHT α-Csn revealed two partially resolved bands at approximately 24 and 25 kDa that corresponded to the α_s1_- and α_s2_-Csn fractions and, surprisingly, although the latter was present in a smaller proportion, it was the most prominent with the stain-free technology we used to reveal proteins (Fig. 3a, line 12). The β-Csn monomer binds more SDS molecules than the α_s1_-Csn monomer and therefore has a higher electrophoretic mobility, even though it is the larger molecule^21^ with a molecular weight of approximately 23 kDa (Fig. 3a, line 15). The control UHT κ-Csn was separated into two main bands localized at approximately 19 and 20 kDa. The κ-Csn dimers were visible at approximately 40 kDa (Fig. 3a, line 18). Total casein monomers appeared as a thick 23–24 kDa band, corresponding to the major α- and β-Csn constituent fractions (Fig. 3a, line 8).

**Figure 4:**
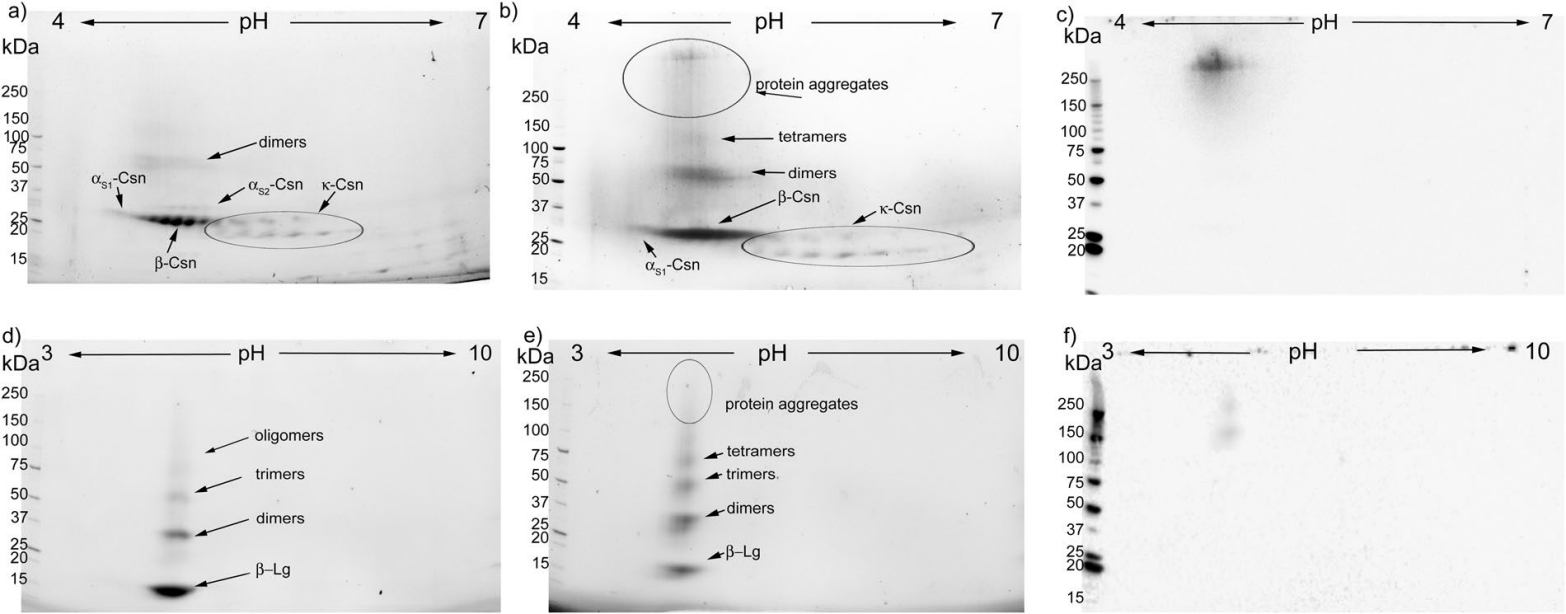
Representative 2-DE gels of TCsn and β-Lg subjected to UHT treatment in the absence (**a** and **d**) or presence of reducing sugar mix (**b** and **e**). Western blots revealing CML formation (**c** and **f**) corresponding to (**b**) and (**e**) gels. In samples without reducing sugars, no CML signal was detected (blots not shown). All samples were stored at room temperature for a period of 90 days. The vertical axis shows the apparent molecular mass in kDa and the horizontal axis shows the pH range. The experiments were performed in triplicate (n = 3).

Interestingly, a slight decrease in monomer mobility was observed in all the glycated samples, including BSA, independent of the UHT treatment. Notably, compared to glycation with lactose alone, the reducing sugar mix produced a more pronounced decrease in mobility (Fig. 3a, lines 2–3, 5–7, 9–11, 11, 13, 16, 17 and 19). This phenomenon might be explained by the formation of Amadori compounds on the polypeptide chains, which would contribute to a slight increase in molecular weight^35^. In glycated proteins, monomer bands (Fig. 3a, lines 2–3, 5–7, 9–11, 11, 13, 16, 17 and 19) were less visible than the controls (Fig. 4a, lines 1, 5, 9, 12, 15), suggesting that a part of the monomer population was aggregated. Concurrently, protein dimers observed in the case of the control caseins were more visible in the lactose-glycated samples and demonstrated slight molecular weight increases (Fig. 3a, lines 2, 5, 9, 13, 16 and 19). Moreover, in the case of UHT treated α- and κ-Csn glycated with the reducing sugar mix, the monomers completely disappeared (Fig. 3a, lines 14 and 20). Also, a tendency to form high-molecular-weight aggregates began with lactose-glycated samples and peaked in caseins glycated with the reducing sugar mix (Fig. 3a, lines 10, 11, 14, 17 and 20).

Similar results were reported in reconstituted milk, since heating in the presence of lactose favours both cross-links products via the Maillard reaction and the formation of dehydroalanine bonds^37^. In the case of α- and κ-Csn glycated with the reducing sugar mix under UHT, protein aggregation was most pronounced. As a result, samples were unable to penetrate the stacking gel (Fig. 3a, lines 14 and 20), despite the strong denaturing and reducing conditions.

In conditions mimicking UHT milk processing, the β-Lg fraction had the least tendency to form aggregates, as demonstrated by the FPLC chromatogram and SDS-PAGE profiles. Other studies that used prolonged exposure to high temperatures (90 °C, up to 24 hours) suggest that glucose retards the aggregation of β-Lg^33^. However, the results indicate that this is not the case under our experimental conditions. The SDS-PAGE profiles of UHT treated TCsn samples glycated with the reducing sugar mix were similar to the non-UHT treated ones, which suggests that the 90 day storage at room temperature heavily contributes to the formation of large molecular aggregates. This indicates that aggregates were formed mainly through glycation cross-links rather than thermal effects, hydrophobic interactions or disulphide bridges, emphasizing the powerful effect of the glucose-fructose syrup to induce severe protein cross-linking in UHT flavoured milk products.

The hypothesis that dietary CML, a non-cross-linking compound, might be able to induce adverse health effects by binding RAGE and increasing inflammatory markers and oxidative stress^38^ motivated this investigation of the formation of CML in glycated milk proteins. CML western blots revealed that non-UHT BSA glycated with lactose or reducing sugar mix had CML-positive bands corresponding to glycated monomers, dimers and trimers with 150–250 kDa molecular weights (Fig. 3b, lines 2 and 3). Interestingly, although β-Lg and BSA are both globular proteins, the glycated β-Lg monomers had no CML signal, regardless of the glycation conditions (Fig. 3b, lines 5–7). Furthermore, this behaviour was observed in all caseins, as CML bands were only detected at molecular weights greater than 150 kDa, corresponding to oligomer and multimer populations (Fig. 3b, lines 10, 11, 13, 14, 17 and 20), although glycated monomers were evident on the SDS-PAGE gels (Fig. 3a, lines 5–7, 9–11, 13, 16, 17 and 19), except for the UHT treated α- and κ-Csn samples glycated in the presence of reducing sugar mix (Fig. 3a, lines 14 and 20). After dispersion in PBS, the casein monomers above the critical micellar concentration likely associated in multimers or micelle-like structures, via hydrogen and hydrophobic bonds^39^, as in milk. The microenvironment at the centre of the structure probably retarded the evolution of Amadori products towards AGEs such as CML. In addition, at approximately 70 °C, the denaturation of β-Lg becomes irreversible via a series of aggregation steps involving intermolecular disulphide bonds, and non-specific hydrophobic, hydrogen and electrostatic interactions^21^. Thus, in β-Lg aggregates, as well as within micelles, some monomers might be shielded from the attack of reducing sugars and the glycation process.

CML western blots revealed that this marker almost exactly overlapped with the low mobility aggregates formed in the glycated BSA (Fig. 3b, lines 2 and 3) and in all the UHT glycated milk protein samples, except for lactose-glycated TCsn, β- and κ-Csn (Fig. 3b lines 5, 6, 10, 13, 14, 17 and 20). However, Meyer *et al*. have shown high-molecular-weight protein aggregates and CML formation on monomers in raw milk subjected to 120 °C for 30 min and for up to 60 min. This study also concluded that α-Csn had the highest susceptibility to aggregation and the formation of CML adducts^40^. Notably, non-UHT TCsn glycated with the reducing sugar mix was also CML positive (Fig. 3b, line 11), while under the same conditions, β-Lg did not present any observable CML formation (Fig. 3b, line 7).

### 2-DE coupled with western blotting for CML analysis of glycation and temperature induced structural protein changes

The 2-DE analysis of the control UHT treated TCsn, stored for 90 days at room temperature, revealed resolved α_s1_-, α_s2_-, β- and κ-Csn fractions (Fig. 4a). The molecular weight of the casein monomer and its pI concurred with that of other studies^5,41^. The α_s1_-Csn fraction was a single, elongated spot and had the most acidic pI, between 4.2 and 4.6, while the α_s2_-Csn fraction was separated into 4 individual horizontal spots, with a pI ranging from 4.8 to 5.1 (Fig. 4a).

β-Csn accounted for the bulk of all the proteins in the TCsn samples and was composed of eight protein sub-fractions, whose pI ranged between 4.6 and 5.1. As discussed for SDS-PAGE, the apparent molecular weight of β-Csn was lower than that of α_s1_-Csn, although its calculated molecular weight was approximately 0.4 kDa larger than that of α_s1_-Csn. The κ-Csn was resolved on a 2-DE and appeared as a series of five horizontal spots at 19 kDa. These were vertically paired with four, 20 kDa heavier spots, as noted in the discussion of the SDS-PAGE results (Fig. 3a, line18). The κ-Csn isoforms had the least acidic pI, ranging from 5.3 to 5.8. In the strong denaturing conditions of 2-DE, the control UHT TCsn profile revealed two horizontally elongated thin spots with molecular weights and pI corresponding to α_s2_-Csn and β-Csn dimers (Fig. 4a). Considering that the sample rehydration buffer contained 8 M urea, 2% CHAPS, and 50 mM dithiothreitol, it is unlikely that these dimers were the result of disulphide cross-linking or hydrophobic and hydrogen bonds. Thus, they are most probably the result of heat-induced dehydroalanine formation and subsequent intermolecular lysinoalanine cross-links^25,37^. UHT TCsn glycated with the reducing sugar mix and stored for 90 days at room temperature lost horizontal resolution for both the α_s2_-Csn and β-Csn fractions, while κ-Csn was mostly preserved (Fig. 4b). In addition, the molecular weights of the monomer spots for α_s1_-, β- and κ-Csn increased, probably due to the formation of Amadori products formed from lactose or hexose sugars, as was confirmed in UHT treated milk samples^5^. In addition, the dimers with a horizontal position on the gel above the α_s2_-Csn and β-Csn monomers were more evident and had an increased molecular weight compared with the dimers observed in the controls (Fig. 4a,b). In a region above the dimers corresponding to 100 and over 250 kDa, there was a poorly resolved heterogeneous mixture of tetramers, oligomers and multimers. The multimers, with pI values ranging between 4.6 and 4.9 and molecular weights over 250 kDa, were readily observable using stain-free revealing technology. The formation of these heterogeneous molecular aggregates in the presence of the reducing sugar mix is explained by AGEs-induced protein cross-linking. Similar results were obtained in UHT milk samples where oligomer formation was shown in the same pI domain, with minor participation of the κ-Csn fraction^5^. However, in that study, 2-DE coupled with MS results could not confirm whether cross-links formed through deamination pathways or through the Maillard reaction. A CML signal was absent in the control TCsn samples (not shown), whereas in the glycated samples, it appeared to overlap with aggregates over 250 kDa formed in the pI range between 4.5 and 5.1, suggesting contributions by α_s1_-, α_s2_- and β-Csn (Fig. 4c). Although CML is a non-cross-linking glycation compound, the overlap demonstrated that the Maillard reaction had a major contribution to the formation of cross-links and these involved proteins that also had CML residues. This confirmed the gel filtration chromatography results and the fluorescence data, which showed that glycated TCsn aggregates were indeed highly fluorescent (Fig. 1a-c).

The control UHT β-Lg 2-DE profile consisted of prominent monomer spots and distinct dimer and trimer spots, all at the same pI of 5.2 (Fig. 4d). In the absence of reducing sugars, the existence of dimers and trimers can only be explained by the formation of heat-induced non-disulphide cross-linking, resistant to the 2-DE denaturing conditions. In glycated β-Lg, protein aggregation increased; dimers, trimers and tetramers were detected in large amounts and there was a vertical, comet-like tail of heterogeneous oligomers (Fig. 4e). This phenomenon consequently diminished the monomer spot. In addition, the monomer, dimer and trimer molecular weights slightly increased as the resolution decreased (Fig. 4e), probably due to lactosylation, as reported in a study on β-Lg in UHT milk^5^, and hexosylation. A CML signal was not detected in the control UHT β-Lg (not shown). However, in the UHT glycated sample, protein aggregates over 150 kDa were CML positive (Fig. 4f). These results emphasize, as in the case of glycated TCsn, that the Maillard reaction contributes to oligomer and multimer formation though non-disulphide cross-links. The fact that monomers, dimers and trimers had no CML despite the increase in molecular weight is indicative that the monomer population had undergone only the first stages of glycation, forming Amadori products, probably due to the predisposition of β-Lg to thermal denaturation, as previously mentioned.

**Figure 5:**
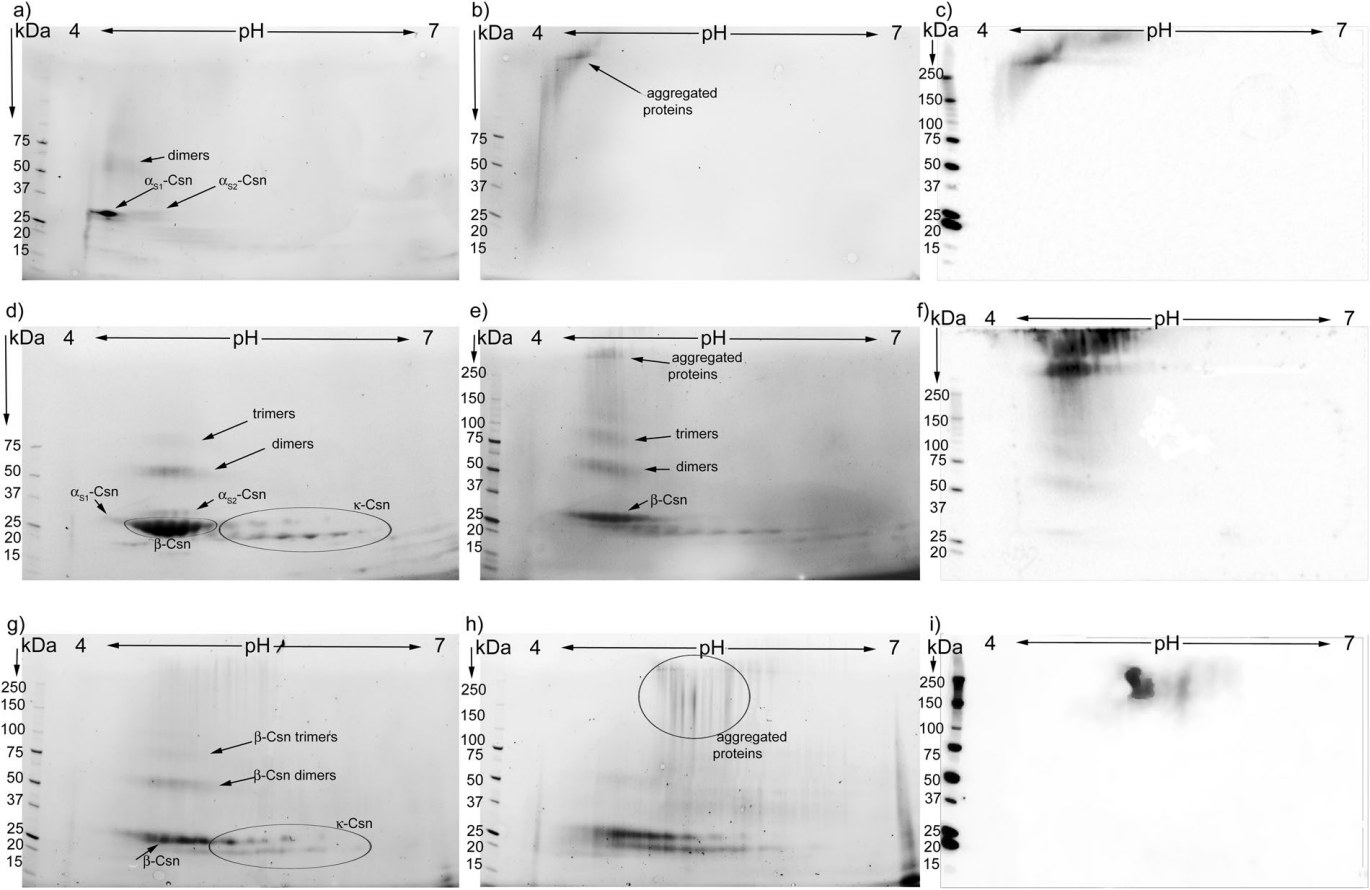
Representative 2-DE gels of α-, β- and κ-Csn subjected to UHT treatment without sugars (**a**,**d** and **g**) and with reducing sugar mix (**b**,**e** and **h**) and corresponding CML blots (**c**,**f** and **i**). In samples without sugars, no CML signal was detected (blots not shown). The vertical axis shows the apparent molecular mass in kDa and the horizontal axis shows the pH interval. All the samples were stored at room temperature for a period of 90 days, as described. The experiments were performed in triplicate (n = 3).

The control α-Csn fraction mainly consisted of an α_s1_-Csn monomer with a pI from 4.2 to 4.6 and a diffuse α_s2_-Csn monomer spot (Fig. 5a). Above the α_s1_-Csn monomer, at 50 kDa molecular weight, a region with a poor resolution indicated dimers. At a molecular weight of approximately 19 kDa, a horizontal smear revealed slight contamination of the α-Csn fraction with κ-Csn (Fig. 5a). The glycated α-Csn 2-DE profile revealed that reducing sugars induced oligomerization of the α_s1_-Csn fraction yielding a heterogeneous aggregate group, which involved all the available monomers (Fig. 5b). Interestingly, the aggregated proteins between 25 to 75 kDa were shifted to the most acidic area of the IPG strip, while the heavier aggregates corresponded to the pI of α_s1_-Csn. While the unglycated control α-Csn was CML-negative (not shown), in the glycated sample the CML signal only coincided with aggregates over 250 kDa, with pI values between 4.2 and 5.5 (Fig. 5c). This wide pI range could be explained by cross-linking between the major α_s1_-Csn fraction, the α_s2_-Csn fraction and the contaminant κ-Csn fraction. According to the pI range of the CML-positive aggregates observed in the glycated TCsn sample, the κ-Csn fractions were minor contributors to aggregate formation, as the κ-Csn protein spots remained largely unaffected (Fig. 4b,c). In the case of glycated α-Csn, at low protein concentrations, according to the amount found in milk, the contaminant κ-Csn was involved in aggregate formation (Fig. 5c).

The control β-Csn 2-DE pattern showed that the β-Csn fraction was contaminated with the other casein fractions, yielding a similar profile to the control TCsn (Figs 4a and 5d). Above the bulk of the β-Csn monomer spot, the four thin spots of α_s2_-Csn, and a spot at approximately 50 kDa corresponding to heat-induced dimers, were noted. The contaminant κ-Csn spot resolution was similar to that described in the control TCsn sample (Figs 4a, 5d). The contamination of β-Csn with the κ-Csn fractions was also observed in the SDS-PAGE analysis (Fig. 3a, line 15). In the control β-Csn sample, only monomer and dimer spots were present, as opposed to the glycated counterpart, where tetramers and heterogeneous molecular weight aggregates appeared. Above 250 kDa, a strong and widespread CML signal covered two pI regions (Fig. 5f): one corresponding to lighter multimers and a pI of 4.6 to 5.1, the range of the β- and α_s2_-Csn fractions, and the other corresponding to heavier aggregates with a pI ranging between 4.5 and 5.5 (Fig. 5f). This unspecific pI range could correspond to aggregates of a mixture of all the contaminant casein fractions. Interestingly, a diffuse CML stain towards the lower molecular weights, down to the dimers and monomers, was observed (Fig. 5f). The pI range shifted slightly towards the acidic domain for the dimer and monomer spots, probably due to the negative charge on the CML carboxyl group (Fig. 5d,e). Regarding the glycation conditions, the β-Csn fraction had a 3-fold diminished protein content compared with that in the TCsn sample, while the concentration of the reducing sugar mix was maintained, which could explain the apparition of the weak CML signal on the glycated β-Csn monomers, dimers and trimers (Fig. 5f).

The control κ-Csn 2-DE profile revealed typical protein spots at pI 5.3 to 5.8 and contamination with the β-Csn monomer and dimers (Fig. 5g). The 2-DE profile of the glycated κ-Csn showed that the monomers lost horizontal resolution, while a faint signal at 37 and 50 kDa, corresponding to glycated κ-Csn and β-Csn dimers appeared. Additionally, CML-positive high-molecular-weight aggregates appeared at pI 5.3 to 5.8 corresponding to the κ-Csn fraction (Fig. 5h,i). The apparent lack of participation of the β-Csn contaminant fraction in aggregate formation can be explained by the detergent-like structure of κ-Csn^21^. It is possible that PBS dispersed κ-Csn to concentrations above the critical micelle concentration^39^. Contamination of the sample with a small amount of β-Csn may have induced the formation of micelle-like structures with the protected β-Csn monomer in the core, enclosed by κ-Csn arranged with its strong hydrophobic N-terminus turned inward and its hydrophilic C-terminal end exposed. Thus, the β-Csn monomers interacted less with the reducing sugars and were glycated to a lesser degree.

**Figure 6:**
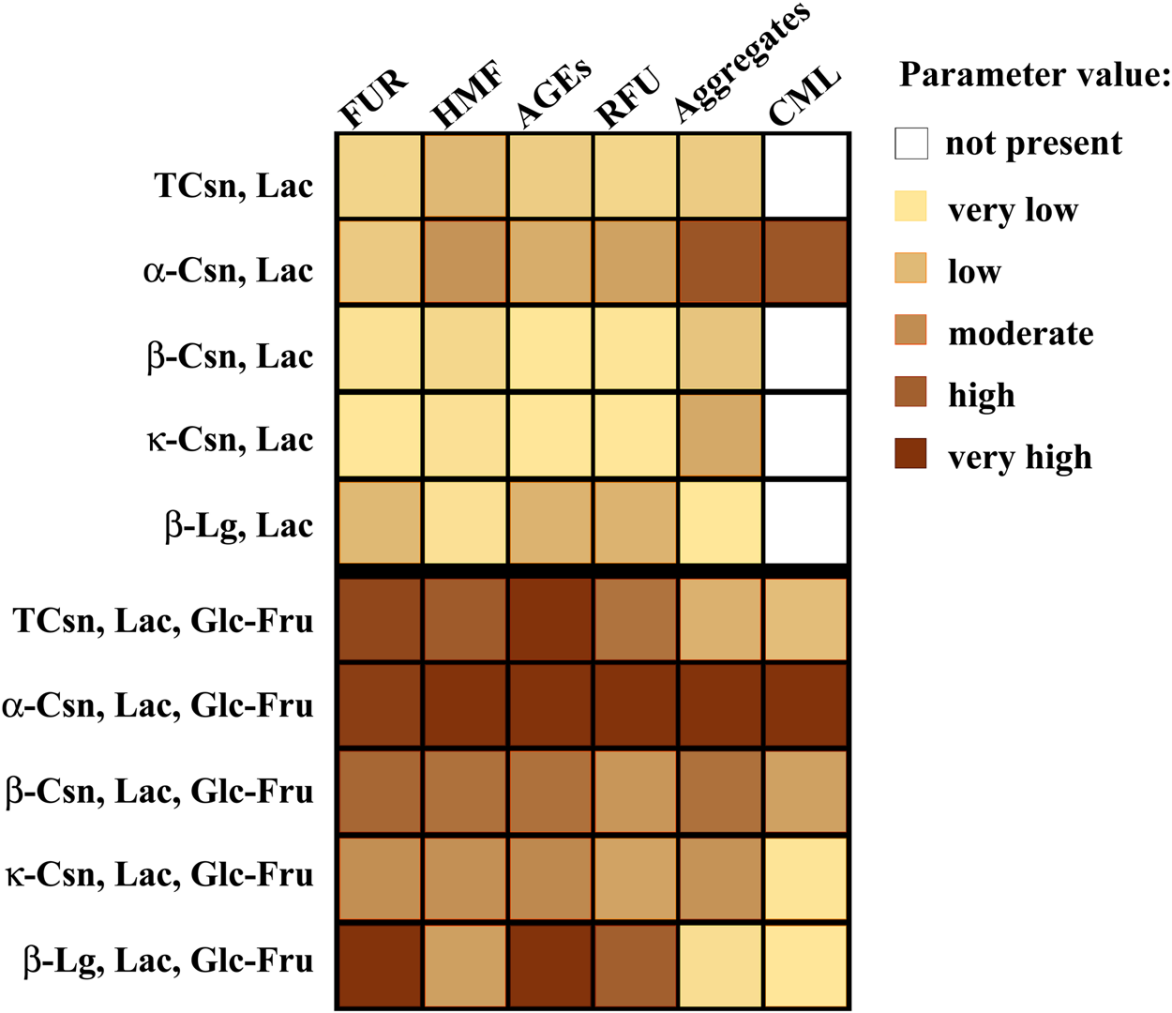
An integrative heat-plot graph, revealing the absolute values of the FUR, HMF, AGEs, relative fluorescence, protein aggregates and CML formation in milk protein samples subjected to UHT treatment, with Lac or Lac, Glc, Fru reducing sugar mix, and subsequently stored for 90 days at room temperature. The value range of each parameter was assigned a colour range, and each value was attributed a corresponding colour code. Data for the protein aggregation represents the results from both FPLC analysis and SDS-PAGE, while the data for CML formation represents the densitometric data from1D western blots.

Both SDS-PAGE and 2-DE coupled with western blot analysis suggested that the majority of CML co-localized with the high-molecular-weight protein aggregates had formed due to the addition of 55 mM glucose-fructose mix to the existing 116 mM lactose (that simulated the presence of glucose-fructose syrup in flavoured milk drinks) (Fig. 6). In addition to the high sucrose content of flavoured milk products, CML dietary intake should be considered a risk factor for diabetes complications and other RAGE-modulated diseases, as CML is the predominant glycation product recognized by RAGE^42^.

Our results show that moderate cross-linking (yielding dimers and trimers) occurred in the milk protein fractions in the absence of reducing sugars, possibly through dehydroalanine formation under UHT conditions. Although CML does not belong to the cross-linking AGEs, only high-molecular-weight aggregates formed in the presence of the reducing sugars, especially the mixture of lactose and glucose-fructose, were co-localized with this advanced Maillard reaction product. The natural tendency of caseins to associate with each other and other proteins^43^, allows for the formation of micelles that create an internal microenvironment shielding the inner monomers and thus delaying glycation. On the outside of the micelle-like structures of casein, the Maillard reaction impacts the exposed proteins, inducing cross-linking and CML formation. This would explain our findings, which showed that glycated monomers and dimers had a slightly increased molecular weight (as described by gel filtration, SDS-PAGE and 2-DE profiles), not because of CML formation (no CML was detected by western blot), but rather due to Amadori compounds. The high susceptibility of α-casein towards glycation is demonstrated by the glycation marker levels, the gel filtration elution and fluorescence fractions patterns, and the electrophoresis coupled with CML immunoblot (Fig. 6).

All milk protein changes reported herein are relevant to ready-to-eat flavoured UHT milk products, as our experiments were performed in conditions similar to the production and storage of such products. Our results show great promise for further use of 2-DE coupled with western blot analysis as a valuable tool to investigate glycation and temperature-induced protein changes in food stuff and more. Our findings strongly suggest that in UHT conditions, prolonged storage effects may cause the protein damage induced by the Maillard reaction.

## Methods

All buffers used were purified using a Chelex-100 resin column (Bio-Rad Laboratories, Hercules, CA, USA) to remove metallic ions.

### Simulation of the UHT process and prolonged storage of flavoured milk products

**Figure 7:**
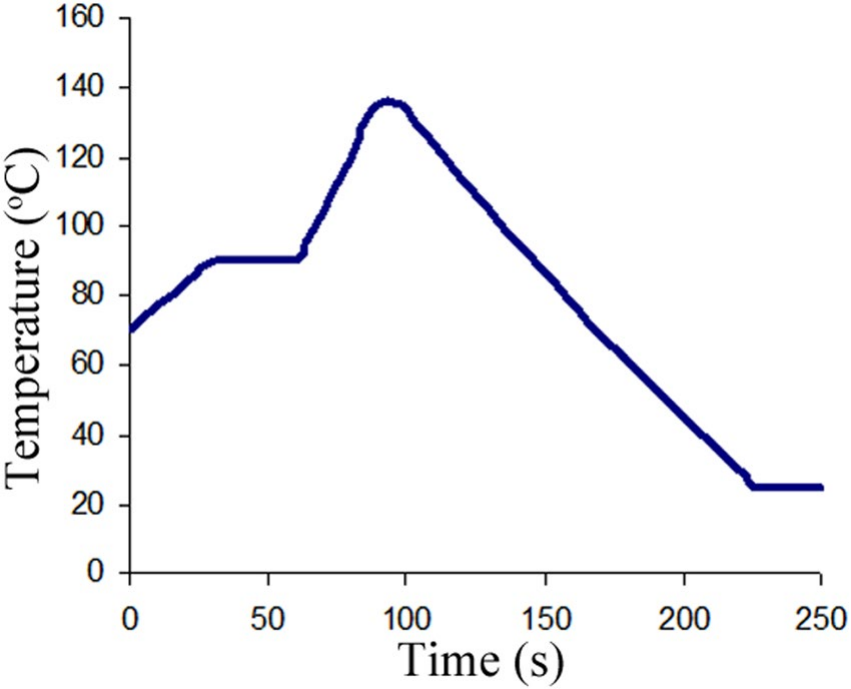
Time-temperature profile of the simulated UHT process.

The milk proteins, 28 mg/mL TCsn, 15 mg/mL α-Csn, 10 mg/mL β-Csn, 3 mg/mL κ-Csn and 1 mg/mL β-Lg, were dispersed in 30 mM PBS, pH 6.8 in the presence or absence of 116 mM lactose or 116 mM lactose and 55 mM glucose-fructose. The suspensions were stirred for 15 min and then sonicated for 30 min. Aliquots of 5 mL were aseptically sealed in glass ampoules and subjected to heat treatment that simulated the heat steps used in the production of flavoured UHT milk. Flavoured milk products are manufactured from reduced-fat milk with the addition of sucrose, approximately 1–3% high fructose or glucose-fructose syrup, different aromas and stabilizers. Then, the mixtures are emulsified at about 60 °C and homogenized at temperatures between 40 and 75 °C^44^. We simulated these industrial heat treatment conditions, beginning with a heating step at 70 °C for 30 min in a water bath, to reproduce the emulsification process. Afterward, the ampoules were rapidly transferred to a second water bath at 90 °C for 30 s to simulate the UHT preheating step and then to a dry bath (Digital Dry Bath Heating Block, Bio-Rad Laboratories, Hercules, CA, USA) preset at 135 °C. The time-temperature profile of the samples was monitored by a thermocouple located in a vial with similar contents. When the temperature reached 135 °C, the samples were incubated for 8 s and immediately returned to the water bath at 70 °C and then cooled. The whole heat treatment lasted less than 250 s, similar to an indirect UHT process (Fig. 7).

All samples were subsequently incubated at 25 °C for 90 days. Thereafter, unreacted reducing sugars were removed by filtration using 10 kDa cut-off membrane filter units (Millipore, St. Charles, MO, USA), according to the manufacturer’s instructions. The filtrates were used to assess the HMF content and the sugar-free protein samples were aliquoted and subsequently frozen at −80 °C.

### FUR and HMF determination

The quantification of both Maillard reaction markers was done using an HPLC system (Dionex UltiMate 3000, with the following modules: SR-3000 Solvent Rack, LPG-3400 SD Pump, WPS-3000SL Autosampler, TCC-3000 SD Column Compartment, DAD-3000 RS Diode Array Detector, FLD-3400RS with Dual-PMT Fluorescent detector and AFC-3000 Automated Fraction Collector, Thermo Scientific, Rockford, USA) and a Chromeleon data acquisition system (Dionex version 7.0.1.272).

### FUR quantification

The protein acid hydrolysis was performed in vacuum hydrolysis tubes (10 × 150 mm, Thermo Scientific, Rockford, USA). Aliquots of 1 mg protein samples were hydrolysed for 23 h at 110 °C under vacuum in the presence of 6 N HCl (Sigma-Aldrich, St. Louis, USA), at a ratio of 2.5 μg of protein per 1 μL of 6 N HCl. The hydrolysed samples were centrifuged at 2,000 × g for 5 min and the supernatants were diluted with ultra-pure water to obtain a 3 N HCl final concentration. Aliquots of 400 μL were concentrated to dryness. The residues were dissolved in 300 μL of ultra-pure water and filtered through a 0.45 μm syringe-tip filter.

Samples of 100 μL were resolved using a Supelcosil LC-8 column (25 cm × 4.6 mm ID, 5 μm particles, Sigma-Aldrich, St. Louis, USA) at 24 °C and a flow rate of 2 mL/min using an isocratic elution with 60 mM sodium acetate, pH 4.3 and a detection wavelength of 280 nm^45^. An external calibration curve in a concentration range from 0.1 to 30 μg/mL FUR HCl salt (PolyPeptide Laboratories, France) was used.

### Free HMF extraction and quantification

were performed according to a previously described protocol^46^, adapted to our conditions. Samples were prepared by mixing 0.5 mL of filtrate, obtained using the 10 kDa cut-off membrane filters, and 1 mL of ethyl acetate in a glass tube, followed by vigorous homogenization for 1 min. This mixture was then allowed to separate into layers. The upper layer was transferred to a clean tube. An additional 1 mL of ethyl acetate was added to the remaining layer, and the procedure was repeated. A volume of 200 μL of 1.5% sodium carbonate solution was added to the combined ethyl acetate layers and the mixture was vigorously homogenized. After separation, the upper layer was transferred to a clean tube and 0.1 g of sodium sulphate was added. After decantation, the ethyl acetate extract was transferred to a clean tube and dried under a gentle stream of nitrogen gas at 40 °C. The residue was dissolved in 0.5 mL of ultra-pure water adjusted to pH 4 with acetic acid. Samples of 100 μL were separated using a reverse-phase column (Acclaim 120 C18, 4.6 × 250 mm, 5μm, 100 Å, non-end-capped, 16% carbon, Thermo Scientific Rockford, USA) at 24 °C and a flow rate of 1 mL/min. The isocratic elution was performed with 90% ultra-pure water and 10% acetonitrile with a detection wavelength of 276 nm. For the HMF standard curve, a sample of 200 μL of 0.2 μg/μL HMF (Sigma-Aldrich, St. Louis, USA) in ethyl acetate was dried under a gentle stream of nitrogen gas at 40 °C. The residue was dissolved in 200 μL of ultra-pure water adjusted to pH 4 with acetic acid. Standard HMF working solutions ranged in concentration from 0.5 to 0.0078 μg/mL.

### Size exclusion chromatography

A fast protein liquid chromatography **(**FPLC) automated system (ÄKTA FPLC, Amersham Pharmacia Biotech Inc., MA, USA) with a Superdex 200 HR 10/30 size exclusion column were used to evaluate the degree of milk protein aggregation. The column was eluted isocratically with 10 mM PBS, pH 7.4; containing 4 M urea and 5% acetonitrile at a constant flow rate of 0.5 mL/min. Milk protein samples were diluted to 3.5 μg/μL in elution buffer containing 50 mM dithiothreitol and incubated for one hour at room temperature. After filtration with 0.45 μm mini-tip syringe filters, a 100 μL sample was injected. The UV absorption of the protein was automatically recorded at 280 nm and one mL fractions were collected. The void volume (*V*_0_) of the column (7.77 mL) was determined using 1 mg/mL Blue Dextran 2000 (Sigma-Aldrich, St. Louis, USA) eluted in the same conditions. To determine the molecular weight of the eluted protein fractions, we used the Gel Filtration Molecular Weight Markers Kit for Molecular Weights 12,000–200,000 Da (Sigma-Aldrich, St. Louis, USA) and Unicorn 4.00 software. The calibration curve equation was

$$\mathrm{log}\,MW=-\,2.1544\,Kav+2.5757,$$

where

$$Kav=\frac{RV(mL)-{V}_{0}(mL)}{GBV(mL)-{V}_{0}(mL)},$$

*RV* represents the retention volume of protein and *GBV* the geometric bed volume of 24 mL.

### Fluorescence assays

The fluorescence of the collected protein fractions associated with pentosidine-like products and Maillard compounds in general was spectrofluorometrically detected at λ_Ex_335 nm/ λ_Em_410 nm and λ_Ex_370 nm/ λ_Em_440 nm^28–30^ respectively, using a JASCO FP 750 spectrofluorometer. Total relative fluorescence of glycated milk proteins at 1 mg/mL was also measured as previously described^36^.

### Immunochemical assays

The total AGEs content of glycated milk proteins was evaluated as previously described^47^ using Advanced Glycation End Product ELISA Kit (Cell Biolabs, San Diego, CA, USA).

### SDS-PAGE coupled with western blotting

Milk proteins (20 μg) were resolved on Criterion TGX Stain Free 4–15% precast gels (Bio-Rad Laboratories, Hercules, CA, USA) at constant 200 V for 45 min. The protein band signal and the molecular weights were evaluated using the ChemiDoc MP System (Bio-Rad Laboratories, Hercules, CA, USA), Image Lab software (version 5.2.1) and Precision Plus Protein WesternC Standards (Bio-Rad Laboratories, Hercules, CA, USA) were used as molecular weight markers. The separated proteins were transferred onto 0.2 μm PDVF membranes (Bio-Rad Laboratories, Hercules, CA, USA) using V3 Western Workflow (Bio-Rad Laboratories, Hercules, CA, USA) and digitized using the ChemiDoc MP System. The membranes were blocked using 5% non-fat dry milk for 2 hours and CML was revealed with OxiSelect CML Immunoblot Kit (Cell Biolabs, San Diego, CA, USA) and Clarity Western ECL Substrate (Bio-Rad Laboratories, Hercules, CA, USA). The chemiluminescence signal was detected using the ChemiDoc MP System.

### 2-DE coupled with western blotting

Samples of 250 μg of milk protein solubilized in a rehydration buffer provided in the ReadyPrep 2-D Starter Kit (Bio-Rad Laboratories, Hercules, CA, USA) were loaded onto pH gradient 11 cm IPG gel strips (pH 4–7 for casein proteins and pH 3–10 for β-Lg; Bio-Rad Laboratories, Hercules, CA, USA), by passive rehydration for 4 h and active rehydration for 10 h at 50 V. Isoelectric focusing of the strips was performed using the Protean IEF Cell (Bio-Rad Laboratories, Hercules, CA, USA) at 20 °C using a 3 step protocol (250 V, 20 min and linear ramp; 8,000 V, 2.5 h, linear ramp and 8,000 V; 2.5 h, 20–30 kV-h, rapid ramp). The strips were equilibrated for 10 min each in equilibration buffer I and II provided in the ReadyPrep 2-D Starter Kit and finally in Tris-glycine-SDS running buffer. The equilibrated strip and the molecular weight marker Precision Plus Protein Standard Plug were added to the top of a Criterion TGX Stain-Free Precast IPG + 1Well Gel, overlaid with agarose and resolved in two dimensions. After 2-DE gel imaging, the gels were further subjected to western blot analysis, performed in the same conditions described in the previous section.
